# Changing Balance of Spinal Cord Excitability and Nociceptive Brain Activity in Early Human Development

**DOI:** 10.1016/j.cub.2016.05.054

**Published:** 2016-08-08

**Authors:** Caroline Hartley, Fiona Moultrie, Deniz Gursul, Amy Hoskin, Eleri Adams, Richard Rogers, Rebeccah Slater

**Affiliations:** 1 Department of Paediatrics, University of Oxford, Oxford OX3 9DU, UK; 2 Nuffield Department of Anaesthetics, John Radcliffe Hospital, Oxford OX3 9DU, UK

## Abstract

In adults, nociceptive reflexes and behavioral responses are modulated by a network of brain regions via descending projections to the spinal dorsal horn [1]. Coordinated responses to noxious inputs manifest from a balance of descending facilitation and inhibition. In contrast, young infants display exaggerated and uncoordinated limb reflexes [2]. Our understanding of nociceptive processing in the infant brain has been advanced by the use of electrophysiological and hemodynamic imaging [3, 4, 5, 6]. From approximately 35 weeks’ gestation, nociceptive-specific patterns of brain activity emerge [7], whereas prior to this, non-specific bursts of activity occur in response to noxious, tactile, visual, and auditory stimulation [7, 8, 9, 10]. During the preterm period, refinement of spinal cord excitability is also observed: reflex duration shortens, response threshold increases, and improved discrimination between tactile and noxious events occurs [2, 11, 12]. However, the development of descending modulation in human infants remains relatively unexplored. In 40 infants aged 28–42 weeks’ gestation, we examined the relationship between nociceptive brain activity and spinal reflex withdrawal activity in response to a clinically essential noxious procedure. Nociceptive-specific brain activity increases in magnitude with gestational age, whereas reflex withdrawal activity decreases in magnitude, duration, and latency across the same developmental period. By recording brain and spinal cord activity in the same infants, we demonstrate that the maturation of nociceptive brain activity is concomitant with the refinement of noxious-evoked limb reflexes. We postulate that, consistent with studies in animals, infant reflexes are influenced by the development of top-down inhibitory modulation from maturing subcortical and cortical brain networks.

## Highlights

• Noxious-evoked brain activity increases in magnitude across the preterm period
• Maturation of nociceptive brain activity coincides with reflex activity refinement
• This may relate to the emergence of top-down inhibition in human infants

## Graphical Abstract

**Figure:**
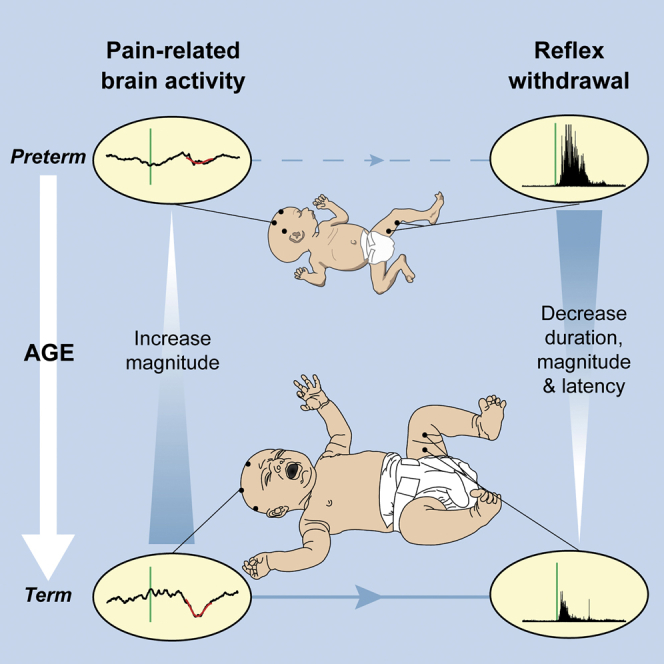

## Teaser

Hartley et al. demonstrate that the maturation of nociceptive brain activity is concomitant with the refinement of reflex withdrawal in human infants. This change in balance of spinal cord excitability and nociceptive brain activity may arise due to the emergence of top-down inhibitory pathways during early human development.

## Results and Discussion

### Magnitude of Nociceptive-Specific Brain Activity Increases with Gestational Age

**Figure 1:**
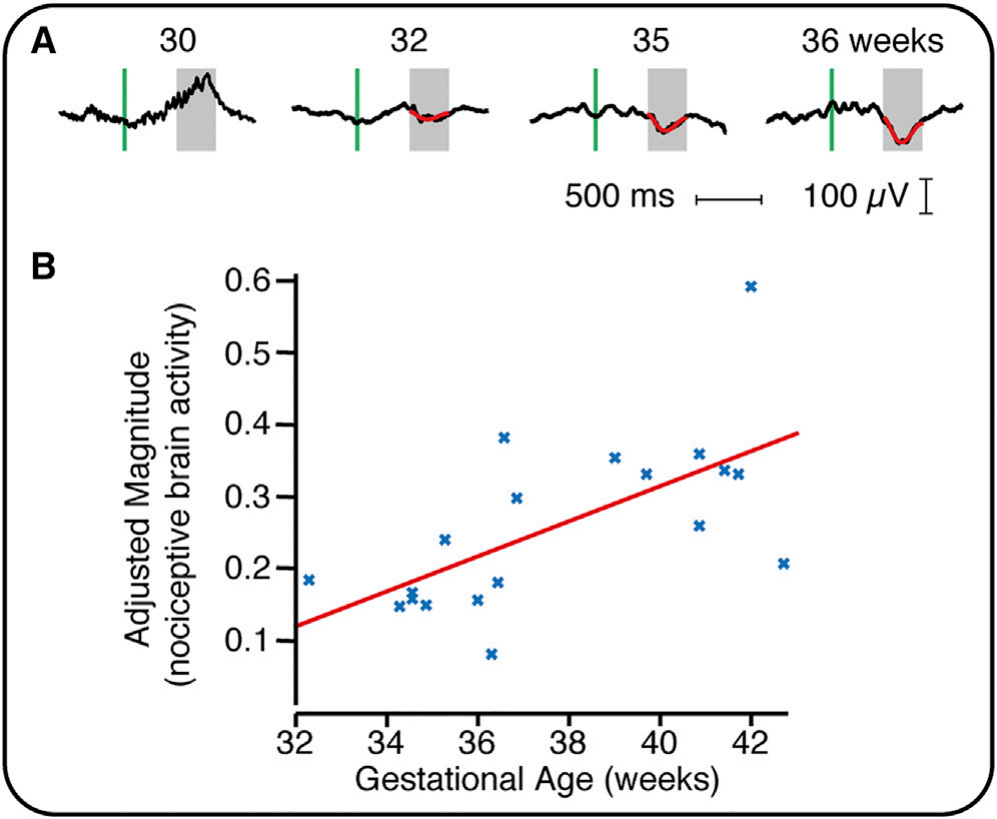
Relationship between Nociceptive-Specific Brain Activity and Gestational Age (A) Four example EEG traces (black lines) from infants at different gestational ages. The green vertical line indicates the point of stimulation, and nociceptive-specific activity is overlaid in red. (B) The magnitude of the nociceptive-specific brain activity significantly increased with gestational age (n = 19). The red line indicates the regression. No significant effect was observed with postnatal age, estimated cumulative prior pain exposure, and previous diagnosis of postnatal infection (Table S1). The magnitude is shown adjusted for these variables and only includes data where nociceptive-specific brain activity has been identified. See also Figure S1.

Electroencephalogram (EEG) responses to a clinically required heel lance were recorded in infants aged between 28 and 42 weeks’ gestation. Nociceptive-specific brain activity (defined as evoked activity distinct from that evoked by non-noxious tactile stimulation—see the Experimental Procedures) was identified at the Cz electrode in 19 out of 36 infants and was not present in any of the infants who were younger than 32 weeks’ gestation (n = 7). In infants who were greater than 32 weeks’ gestation, nociceptive-specific brain activity was identified in 66% of infants (19 out of 29), and the magnitude of this activity significantly increased with gestational age (Figure 1; p = 0.031, regression coefficient β = 0.024, n = 19). This could not be accounted for by postnatal age, estimated cumulative prior pain exposure, or previous diagnosis of postnatal infection, which were included within the statistical model (Table S1).

Figure 1 includes four example EEG responses across a range of gestational ages. In the youngest infants, nociceptive-specific brain activity did not occur and non-modality specific bursts of activity [7, 8, 9, 10], known as delta brushes, could be identified. This supports previous observations that the likelihood of evoking nociceptive-specific patterns of brain activity increases with gestational age and that the brain’s ability to discriminate between tactile and noxious inputs emerges at approximately 35 weeks’ gestation [7].

The emergence of nociceptive-specific brain activity during the preterm period coincides with the disappearance of the subplate and the formation of direct thalamocortical connections [7, 13]. Given that key clinical factors and demographic characteristics have been controlled for, the increase in magnitude of the evoked activity is likely to represent a normal maturational process. It is plausible, for example, that it may reflect the strengthening and elaboration of thalamocortical connectivity that occurs during this developmental period [14].

Here we focus on activity at the Cz electrode as nociceptive-specific brain activity has previously been identified and characterized at this electrode site [6, 7]. In the absence of this activity, responses with similar latency and morphology did not occur at other electrode sites (Figure S1). This does not rule out the likelihood that nociceptive activity could be recorded at other electrode sites, but it provides a useful way of quantifying the maturation of nociceptive processing in the developing infant brain. Furthermore, activity recorded at the Cz site is likely to include contributions from numerous brain regions, including the primary and secondary somatosensory cortices, the anterior cingulate cortex, and the insular cortices [15].

### Nociceptive Reflex Withdrawal Activity Becomes More Refined with Gestational Age

**Figure 2:**
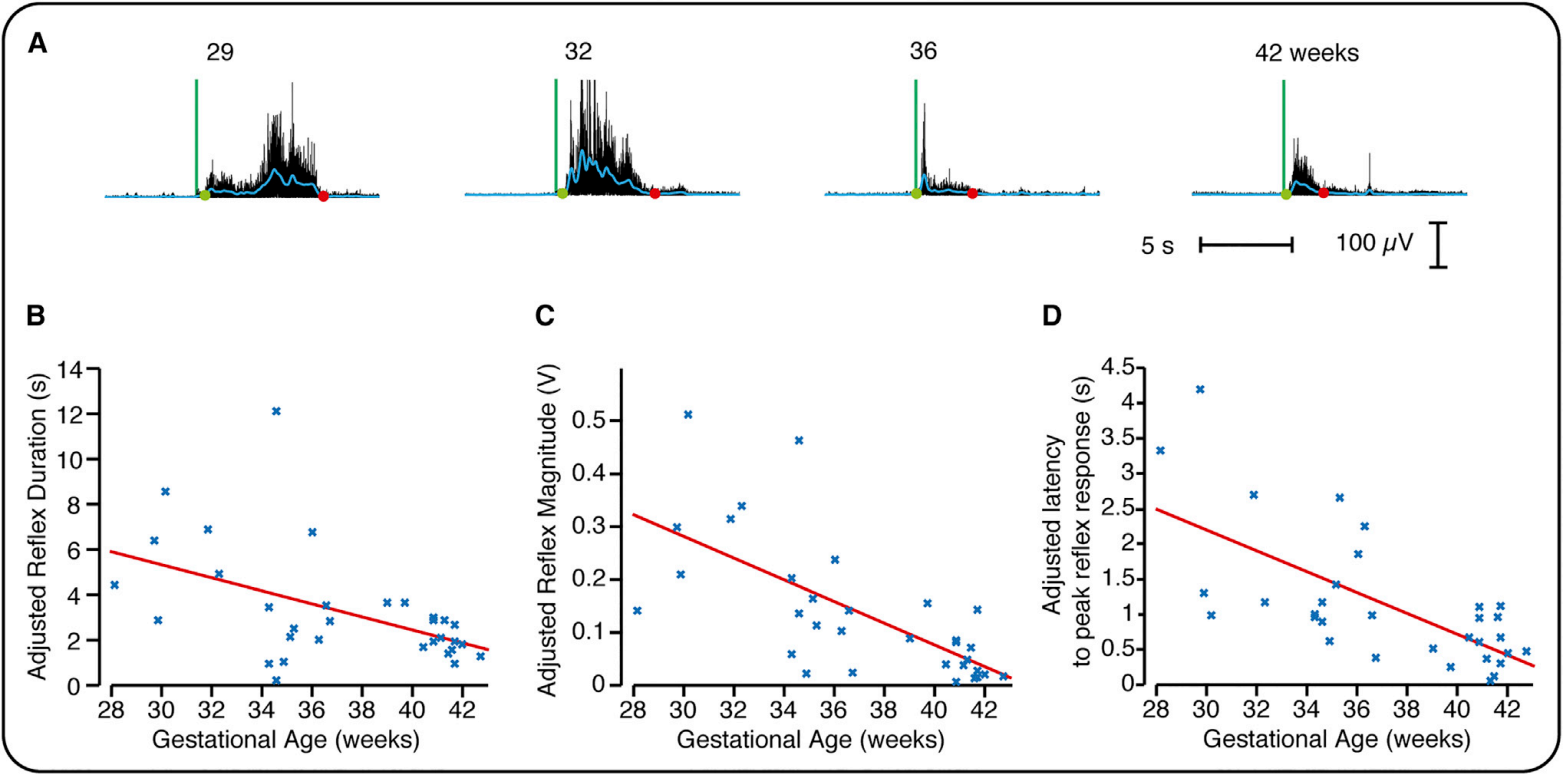
Refinement of Reflex Withdrawal Activity (A) Four example EMG traces (black lines) from infants at different gestational ages. The green vertical line indicates the point of stimulation, and the blue line represents the smoothed reflex withdrawal activity. The start and end points are identified by the green and red dots, respectively (see also Figure S3). (B–D) The duration (B), magnitude (C), and latency to the peak of the reflex withdrawal activity (D) significantly decreased with gestational age (n = 32). No significant effect was observed with postnatal age, estimated cumulative prior pain exposure, and previous diagnosis of postnatal infection (Table S1). The data are shown adjusted for these variables.

Reflex withdrawal activity was recorded in the limb ipsilateral to the site of stimulation by placement of electromyography (EMG) electrodes over the biceps femoris. A novel algorithm that defines the start and end points of infant reflex withdrawal was used to identify the changing characteristics of reflex activity across development (see the Experimental Procedures). Figure 2A includes four example EMG traces across a range of gestational ages. We observed that increasing gestational age was associated with a significant decrease in the duration (Figure 2B; p = 0.039, β = −0.29, n = 32), magnitude (Figure 2C; p = 0.001, β = −0.02), and latency to the peak reflex response (Figure 2D; p = 0.002, β = −0.15; see also Table S1), representing a decrease in magnitude of 20 mV per week and a decrease in duration of 0.3 s per week. This refinement in reflex withdrawal activity is consistent with previous observations in both animals and human infants [2, 16].

Infant limb reflexes are exaggerated and disorganized. In young rat pups, reflexes have longer duration, higher magnitude, and lower response thresholds compared with adult animals [12, 16]. Moreover, rat pups are more likely to inappropriately flick their tail toward a noxious stimulus than away from it [17]. In preterm infants, reflexes are prolonged, thresholds lie within the tactile range, and bilateral responses occur after unilateral stimulation [2, 11, 18]. The lower reflex withdrawal threshold likely allows for activity-dependent development of nociceptive processing [2, 17, 19]. In addition, reflex maturation is dependent on supraspinal activity, with neonatal spinal cord transection resulting in disorganized and exaggerated reflexes in the adult animal [20]. In young rat pups, the cutaneous receptive fields of dorsal horn cells are larger, mature over the first few weeks of postnatal life [21], and contribute to the exaggerated reflex activity observed in young animals [22, 23, 24].

### Refinement of Reflex Withdrawal Activity Is Related to Maturation of the Nociceptive-Specific Brain Activity

**Figure 3:**
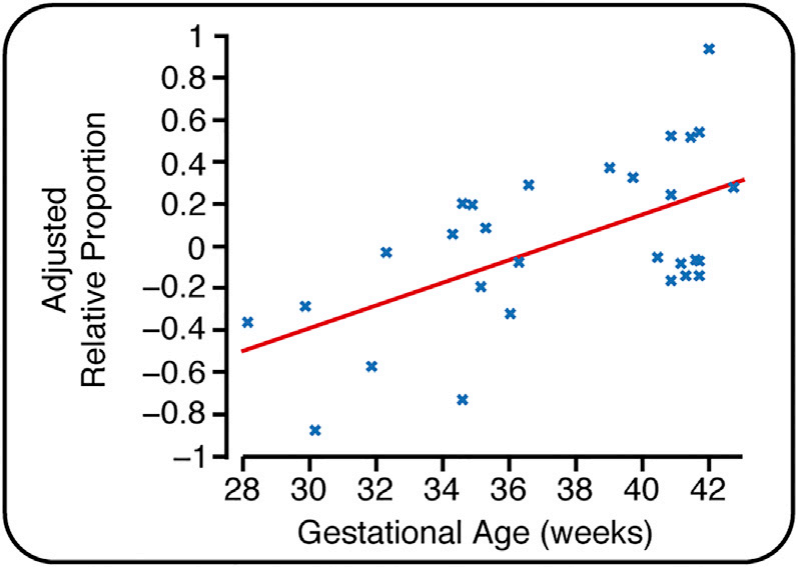
The Relationship between Nociceptive-Specific Brain Activity and Reflex Withdrawal Activity with Gestational Age The relative proportion of brain and spinal cord activity for each infant plotted against gestational age (n = 29; where brain activity and reflex activity were both recorded without artifact). The values of the relative proportion are limited between −1 and 1, where −1 indicates maximal reflex withdrawal (within the population) with no concomitant nociceptive-specific brain activity and 1 indicates maximal nociceptive-specific brain activity with no concomitant reflex withdrawal (see the Experimental Procedures). See also Figure S2 and Table S1.

Finally, we investigated the development of the relationship between nociceptive-specific brain activity and reflex withdrawal activity. When considering the emergence of the nociceptive-specific brain activity and the diminution of the reflex activity, the relative proportion of nociceptive-specific brain activity to reflex withdrawal activity within individual infants significantly increases with gestational age (Figure 3; p = 0.024, β = 0.054, n = 29; see also Table S1). There was no significant effect of postnatal age, estimated cumulative prior pain exposure, or previous diagnosis of postnatal infection (see Table S1). Noxious input in younger infants elicits more prolonged reflex withdrawal activity but comparatively less nociceptive-specific brain activity compared with older infants. The presence of more mature nociceptive-specific brain activity in older infants is concomitant with a more acute reflexive response (Figure 3). All electrophysiological recordings in which both EMG and EEG activity were artifact-free are included in Figure S2.

Independent observations describing the reduction in reflex withdrawal activity [2] and the emergence of nociceptive-specific brain activity [7] across the preterm period have been reported. In term infants, the cortical and subcortical brain regions activated by noxious stimulation are similar to those seen in adults [4], and there is a clear correlation between the magnitude of noxious-evoked brain activity and spinally mediated reflex withdrawal activity [25]. However, the emerging relationship between spinally mediated reflexes and nociceptive brain activity that underpins the perception and expression of pain has not previously been investigated in premature infants. The change in balance of nociceptive-specific brain activity and spinally mediated reflex activity suggests that the maturation of nociceptive brain activity may facilitate the inhibitory modulation of spinal nociceptive circuitry. This is consistent with animal studies; in rat pups, the descending control system is immature and the drive from the rostral ventral medulla is predominantly excitatory [26]. This contributes toward the exaggerated and uncoordinated reflex activity observed in young animals [12, 17, 22, 26, 27]. Descending inhibitory influences develop over the first few postnatal weeks, and from the fourth postnatal week more mature cerebral processing of nociceptive input can dampen spinally mediated reflex withdrawal activity [26]. Additionally, it has recently been proposed that the descending facilitation of spinal nociception that is observed in the first few postnatal weeks is likely to be generated by spontaneous brainstem activity that is independent of sensory input [28].

In adults, top-down connections play a key role in modulating pain perception [1]. This involves an extensive network of brain regions that include the anterior cingulate cortex, insular cortices, and brainstem [1]. In full-term infants, these brain regions are actively involved in processing nociceptive input [4] and may contribute toward the generation of the electrophysiological nociceptive activity characterized here [15]. We postulate that the maturation of cortical networks, which is reflected here as an increase in the magnitude of nociceptive-specific brain activity, contributes to the emergence of top-down inhibitory modulation of spinal nociceptive circuitry. Analysis of fMRI data should be used to explore how regional brain development modulates pain-related behavior in human infants.

### Conclusions

In conclusion, we demonstrate that across the early developmental period from 28–42 weeks’ gestation, the maturation of noxious-evoked brain activity coincides with the refinement of reflex withdrawal activity. We postulate that the change in balance of nociceptive-specific brain activity and reflex withdrawal with gestational age is driven by the development of supraspinal processing, which results in the modulation of spinally mediated reflex withdrawal. This study provides new insights into the functional development of neural pathways that underlie human pain behavior and represents an important step toward translation of laboratory animal data.

## Experimental Procedures

### Subjects

40 infants were recruited between May 2012 and June 2015 from the Neonatal and Maternity Units of the John Radcliffe Hospital, Oxford. Infants were aged between 28 and 42 weeks’ gestation at time of study and aged 33 days or less. Further infant demographics are listed in Tables 1 and S2. (See “Infant Eligibility” in the Supplemental Experimental Procedures.) Ethical approval (National Research Ethics Service) was obtained, and informed written parental consent was gained prior to each study. The study was carried out in accordance with the standards set by the Declaration of Helsinki and Good Clinical Practice guidelines.

### Experimental Protocol

All heel lances performed in the study were clinically required as part of the infant’s medical care. EMG and EEG activity was recorded during a background rest period and during a clinically required heel lance (see “EEG and EMG Acquisition” in the Supplemental Experimental Procedures).

### Analysis

#### EEG

EEG was filtered 0.5–70 Hz, with a notch filter at 50 Hz. 1500 ms epochs were extracted with 500 ms before the stimulus and traces were baseline corrected to the pre-stimulus mean. A total of four infants were rejected from EEG analysis because epochs contained gross movement or signal artifacts (for example, one epoch was rejected due to repetitive artifacts caused by the infant’s respiratory support).

Nociceptive-specific brain activity is known to be evoked at the Cz electrode [6]. For calculation of the magnitude of the activity, the component of nociceptive-specific brain activity (defined in an independent sample of term infants [25]) was projected onto the data using singular value decomposition [25]. This enabled the weights (magnitude) of the evoked nociceptive-specific brain activity to be determined in this study. The EEG traces were Woody filtered, with a maximum jitter of ±50 ms, in the region of 400–700 ms after the stimulus, by identifying the maximum correlation with the component of nociceptive-specific brain activity. The weights of the nociceptive-specific component (which are a reflection of the magnitude of the nociceptive-specific brain activity within an individual response) were then calculated in the region 400–700 ms after the stimulus at the Cz electrode. A threshold to define whether nociceptive-specific brain activity was present was set by comparison of the weights of the evoked activity to the weights generated from background brain activity (see “Threshold for Nociceptive-Specific Brain Activity” in the Supplemental Experimental Procedures).

#### EMG

Epochs were extracted from 15 s before to 15 s after the stimulus, and the signals were filtered between 10 and 500 Hz with a notch filter at 50 Hz (and harmonics) and rectified. Epochs were rejected due to high signal levels from high impedances and electrocardiogram (ECG) artifacts. For accurate identification of the start and end times of the reflex, a novel algorithm was developed (see “Determination of EMG Characteristics” in the Supplemental Experimental Procedures and Figure S3). Five infants were excluded from EMG analysis due to artifact. A further three infants were excluded as in two cases no end point was identified by the algorithm and in one case no reflex withdrawal could be identified.

#### Comparison of Brain Activity and Reflex Withdrawal

The maturation of brain activity and reflex withdrawal was compared by examining the relative proportion of the two signals across the gestational age range. This was determined by first calculating the proportion of each signal for each infant. This was defined as the signal normalized by the maximal signal (across all infants). So for each infant, the duration of the reflex withdrawal was divided by the maximum duration across the population, and the weight of the nociceptive-specific brain activity was divided by the maximum weight across the population. Below-threshold brain activity responses were set to 0 as the evoked activity was not greater than the levels of spontaneous activity observed in the background data. The relative proportion was then calculated as the difference in the proportion of nociceptive-specific brain activity and the proportion of reflex withdrawal. This gives a value between −1 and 1, where −1 indicates a maximal reflex withdrawal coupled with no nociceptive-specific brain activity and 1 indicates maximal nociceptive-specific brain activity with no reflex withdrawal.

#### Cumulative Prior Pain Exposure and Previous Diagnosis of Postnatal Infection

To provide an estimate of cumulative prior pain exposure, we retrospectively reviewed each infant’s electronic and paper clinical records. The total number of aspirations (oropharyngeal or endotracheal) and tissue-damaging procedures performed for blood taking (including heel lances, venepuncture, and intravenous cannulations) from time of birth to time of study were documented. These procedures were chosen as they feature among the top six most common painful procedures performed in the first 2 weeks of life in infants receiving intensive care, as estimated by a large multicenter prospective study [29], and are well documented by the clinical team, facilitating retrospective review.

Infants with a previous diagnosis of postnatal infection were established by review of clinical records to identify infants who had received treatment for suspected culture-negative sepsis.

#### Statistical Analysis

Statistical analysis was performed in MATLAB R2014b (MathWorks). Linear regression analysis was conducted with gestational age at study, postnatal age, estimated cumulative prior pain exposure, and previous diagnosis of postnatal infection included as independent variables. Normality of the residuals was confirmed using Q-Q plots. Regression figures are presented with the dependent variable adjusted for postnatal age, estimated cumulative prior pain exposure, and previous diagnosis of postnatal infection.

## Author Contributions

R.S., F.M., and E.A. designed the study; F.M., C.H., and A.H. performed the experiments; C.H., F.M., R.S., and D.G. analyzed the data; C.H., F.M., and R.S. wrote the paper; and R.S., C.H., F.M., D.G., R.R., A.H., and E.A. made a significant contribution to data interpretation. All authors critically revised the manuscript.

## Figures and Tables

**Table 1:** Description of Infant Demographics

Demographic Details	Values
Gestational age at birth (weeks)—median (IQR)	34.4 (29.6–40.6)
Gestational age at time of study (weeks)—median (IQR)	36.4 (33.3–40.9)
Postnatal age at time of study (days)—mean (SD)	12.1 (11.1)
Birth weight (g)—median (IQR)	2,194 (1,538–3,627)
Weight at study (g)—median (IQR)	2,325 (1,620–3,627)
Male infants (%)	20 (50)
Multiple gestation infants (%)	7 (18)
Spontaneous vaginal deliveries (%)	18 (45)
Assisted/caesarian deliveries (%)	22 (55)
Apgar score at 1 min—mean (SD)	7.2 (2.6)
Apgar score at 5 min—mean (SD)	9.1 (1.4)
Infants admitted to NICU (%)	23 (58)
Infants ventilated during admission (%)	9 (23)
Days of ventilation—mean (SD)	4.8 (6.4)
Estimated cumulative prior pain exposure—median (IQR)	8 (3.8–28)
Infants with grade I IVH (%)	3 (7)
Infants with history of previous surgery (%)	2 (5)
Infants with a previous diagnosis of postnatal infection (%)	30 (75)
Infants with history of necrotizing enterocolitis (%)	2 (5)

IQR, interquartile range; NICU, neonatal intensive care unit; IVH, intraventricular hemorrhage. See also Table S2.

## Supplemental Information

Document S1. Supplemental Experimental Procedures, Figures S1–S3, and Tables S1 and S2

Document S2. Article plus Supplemental Information
