# Supplementary material for: Changing Balance of Spinal Cord Excitability and Nociceptive Brain Activity in Early Human Development
Source: Curr Biol. 2016 Aug 8;26(15):1998–2002. doi: 10.1016/j.cub.2016.05.054 (PMC4985558; doi:10.1016/j.cub.2016.05.054)
Supplement: Document S1. Supplemental Experimental Procedures, Figures S1–S3, and Tables S1 and S2 [file mmc1.pdf]

Current Biology, Volume *26*

Supplemental Information

Changing Balance of Spinal Cord Excitability and Nociceptive Brain Activity in Early Human Development

Caroline Hartley, Fiona Moultrie, Deniz Gursul, Amy Hoskin, Eleri Adams, Richard Rogers, and Rebeccah Slater

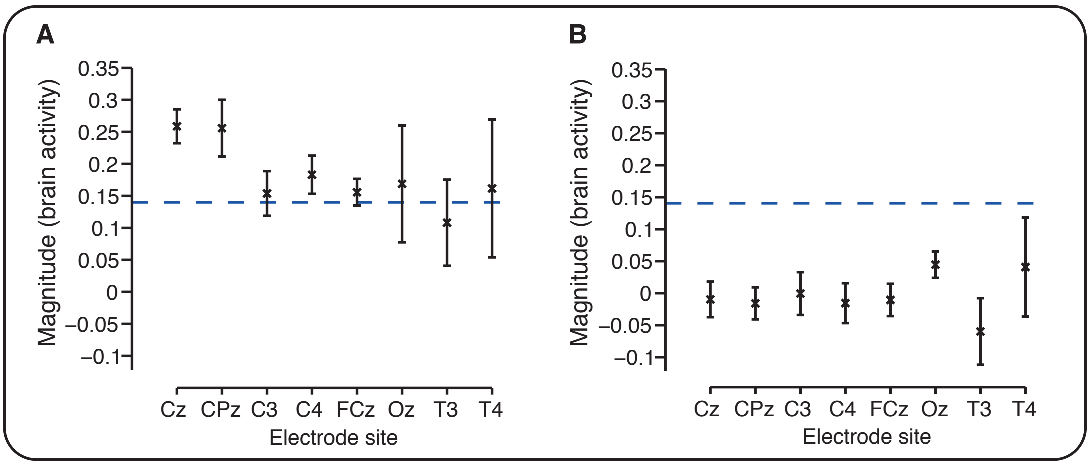

Figure S1: Magnitude of the response at other electrode sites, related to Figure 1. Magnitude of the nociceptive brain activity at each electrode site grouped according to whether nociceptive-specific activity was identified at Cz. (A) shows the average activity when nociceptive-specific activity was identified at Cz and (B) shows the average activity when nociceptive-specific activity was not identified at Cz. Error bars indicate the standard error of the mean. Blue dashed lines indicate the threshold for a nociceptive-specific response.

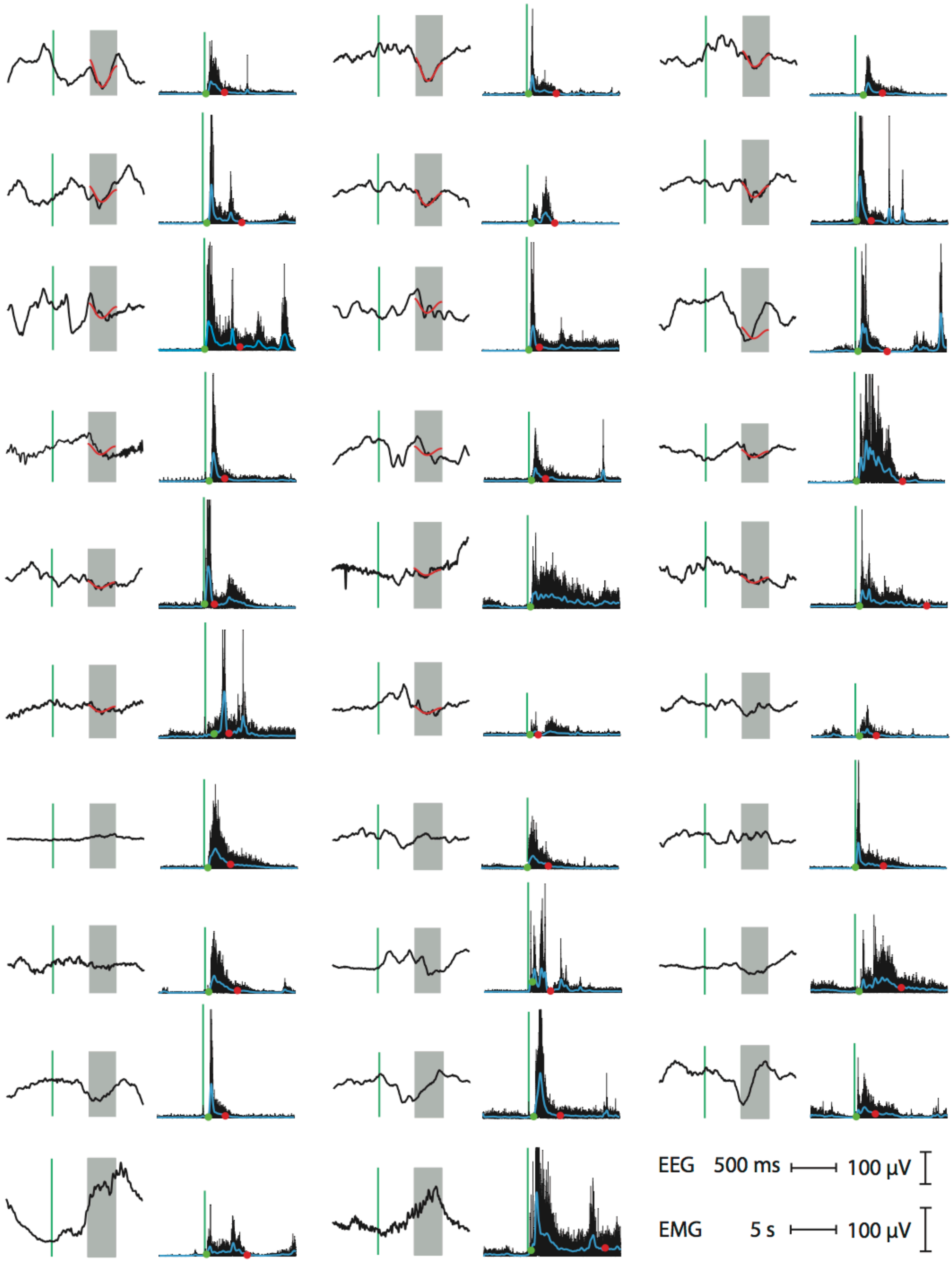

Figure S2: Brain activity and reflex withdrawal activity, related to Figure 3.
Individual infant EEG traces and EMG traces for all infants where artifact-free recordings were simultaneously recorded (3 infants are shown per row; EEG activity at the Cz electrode site is displayed on the left and EMG activity is displayed on the right; n=29). The point of stimulation is indicated by the

green vertical line. The grey box indicates the time region from 400 - 700 ms after stimulation. When nociceptive-specific responses were identified, the component of nociceptive-specific brain activity is overlaid in red. The raw EMG activity is overlaid with the smoothed EMG activity (blue line, see Supplemental Experimental Procedures). The green dot represents the start of the reflex and the red dot represents the end.

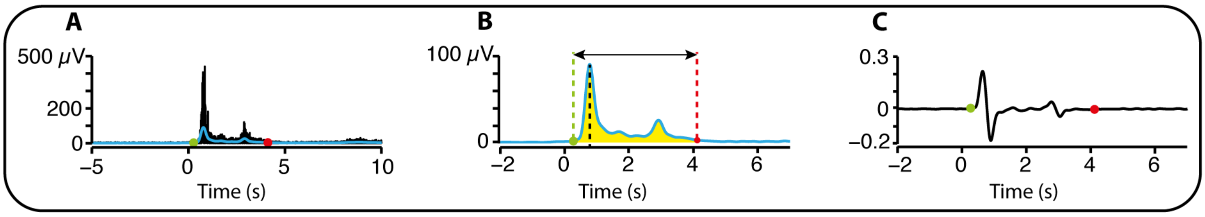

Figure S3: Novel algorithm used to characterise reflex withdrawal, related to Figure 2.
(A) Example reflex withdrawal recorded using EMG (activity shown in black). This activity is smoothed (blue line), and the start (green dot) and end times (red dot) are calculated using a novel algorithm.
(B) The smoothed EMG signal (blue, from A) is used to find the magnitude of the reflex withdrawal, given by the area under the curve between the start and end times (shaded yellow), the duration of the reflex withdrawal, defined as the time between the start and end points (indicated by the arrow), and the peak latency (time to the black dashed line). (C) The differential (black) of the smoothed signal (shown in blue in A, B), which is used to calculate the start (green dot) and end (red dot) times of the reflex withdrawal.

Dependent variables	Gestational Age (at time of study)			Postnatal Age			Previous diagnosis of postnatal infection			Estimated cumulative prior pain exposure		
	**β (CI)**	**β* (CI)**	**P**	**β (CI)**	**β* (CI)**	**P**	**β (CI)**	**β* (CI)**	**P**	**β (CI)**	**β* (CI)**	**P**
Magnitude of nociceptive-specific brain activity (n = 19)	0.024 (0.003 to 0.046)	0.67 (0.07 to 1.27)	**0.031***	0.0004 (-0.007 to 0.008)	0.041 (-0.66 to 0.74)	0.90	-0.092 (-0.23 to 0.041)	-0.80 (-1.95 to 0.35)	0.16	0.0008 (-0.002 to 0.004)	0.18 (-0.44 to 0.80)	0.54
Duration of reflex withdrawal (n = 32)	-0.29 (-0.56 to -0.016)	-0.50 (-0.98 to -0.027)	**0.039***	0.035 (-0.098 to 0.17)	0.15 (-0.43 to 0.73)	0.59	0.55 (-1.86 to 2.96)	0.22 (-0.75 to 1.20)	0.64	-0.028 (-0.071 to 0.015)	-0.33 (-0.84 to 0.18)	0.20
Magnitude of reflex withdrawal (n = 32)	-0.020 (-0.032 to -0.009)	-0.77 (-1.21 to -0.32)	**0.001***	-0.003 (-0.009 to 0.003)	-0.29 (-0.84 to 0.26)	0.28	0.019 (-0.087 to 0.12)	0.16 (-0.75 to 1.07)	0.72	-0.0006 (-0.003 to 0.001)	-0.15 (-0.63 to 0.33)	0.52
Peak latency of reflex withdrawal (n = 32)	-0.15 (-0.24 to -0.060)	-0.72 (-1.16 to -0.30)	**0.002***	-0.031 (-0.075 to 0.011)	-0.40 (-0.93 to 0.13)	0.14	0.27 (-0.51 to 1.05)	0.31 (-0.57 to 1.19)	0.48	0.006 (-0.008 to 0.020)	0.21 (-0.25 to 0.68)	0.35
Relative proportion of EEG to EMG activity (n = 29)	0.054 (0.008 to 0.10)	0.64 (0.09 to 1.18)	**0.024***	0.003 (-0.018 to 0.024)	0.088 (-0.56 to 0.73)	0.78	-0.29 (-0.70 to 0.12)	-0.79 (-1.91 to 0.32)	0.15	0.002 (-0.005 to 0.009)	0.16 (-0.40 to 0.72)	0.56

Table S1: Regression models, related to Figures 1, 2 and 3.
For each of the EEG and EMG characteristics (dependent variables) the regression coefficients (β); standardised regression coefficients (β*); confidence interval limits (CI) and p-values that are associated with the infant characteristics (gestational age, postnatal age, postnatal infection and estimated cumulative prior pain exposure) are given.

Subject	GA at study (weeks)	GA at birth (weeks)	Weight at birth (g)	Apgar score at 5 minutes	NICU	Mechanical ventilation (days)	Time since morphine administered (days)	IVH	PNI	Prior pain	Surgery
**1**	28.1	25.1	920	10	✓	1			✓	32	
**2**	29.7	28.3	855	6	✓		8		✓	16	
**3**	29.9	25.6	805	8	✓	5	28		✓	58	
**4**	30.1	28.9	640	8	✓					13	
**5**	30.9	29.0	1580	8	✓		12	✓		28	
**6**	31.6	27.3	720	8	✓	21	7	✓	✓	299	
**7**	31.7	29.0	1415	9	✓					17	
**8**	31.9	27.7	1315	10	✓	2	19		✓	124	
**9**	32.3	31.9	1580	8	✓				✓	6	
**10**	32.9	28.7	765	5	✓	1				35	
**11**	33.4	29.7	636	9	✓					41	
**12**	34.3	29.4	1390	4	✓	7	18		✓	111	Bowel surgery
**13**	34.3	32.0	2340	9	✓				✓	5	
**14**	34.6	31.0	1800	8	✓			✓	✓	8	
**15**	34.6	31.1	1610	10	✓				✓	14	
**16**	34.9	33.0	1820	9	✓	2	12		✓	28	
**17**	35.1	31.9	2020	8	✓	2	13		✓	42	Bowel surgery
**18**	35.3	31.9	1982	10	✓					14	
**19**	36.0	32.3	1626	10	✓				✓	16	
**20**	36.3	35.9	2360	10						5	
**21**	36.4	36.1	1910	10	✓					8	
**22**	36.6	33.0	2048	9	✓	2	20			35	
**23**	36.7	36.0	2440	10	✓					14	
**24**	36.9	36.1	3330	10	✓				✓	23	
**25**	39.0	38.9	3215	10					✓	2	
**26**	39.7	39.3	3595	10					✓	2	
**27**	40.4	40.1	5120	10					✓	4	
**28**	40.9	40.1	3400	10					✓	4	
**29**	40.9	40.3	3770	10					✓	5	
**30**	40.9	40.9	3500	10					✓	1	
**31**	41.0	40.6	3360	10					✓	3	
**32**	41.1	41.0	3925	10					✓	1	
**33**	41.3	41.1	3975	10					✓	1	
**34**	41.4	41.3	4645	9					✓	1	
**35**	41.6	40.7	3725	10					✓	4	
**36**	41.7	41.4	4220	10					✓	4	
**37**	41.7	41.6	3955	10					✓	1	
**38**	41.7	41.1	4140	10					✓	3	
**39**	42.0	41.7	4320	10					✓	1	
**40**	42.7	42.0	3464	8					✓	4	

Table S2: Individual infant demographics, related to Table 1.
GA – gestational age, NICU – indicates whether the infant was admitted to the neonatal intensive care unit, PNI –postnatal infection: indicates whether the infant had received treatment for suspected culture-negative sepsis prior to the study, prior pain – indicates the estimated cumulative prior pain exposure.

Supplemental Experimental Procedures

Infant Eligibility

Infants were not eligible for inclusion if they had documented neurological malformations or symptoms, genetic disorders, or a history of meningitis, culture-positive sepsis, or maternal substance abuse. Clinical cranial ultrasound reports were reviewed for all infants born at <32 weeks gestation or weighing <1500g at birth. Only infants with normal scans or a small, uncomplicated, unilateral, isolated subependymal haemorrhage (grade 1 IVH; [S1]) were included. At the time of study, all infants were haemodynamically stable, not requiring mechanical ventilation, and had not received analgesics or sedatives in the preceding seven days.

EEG and EMG Acquisition

Electrophysiological activity was acquired using the SynAmps RT 64-channel headbox and amplifiers (Compumedics Neuroscan), with a bandwidth from DC - 400 Hz and a sampling rate of 2 kHz, and recorded using CURRYscan7 neuroimaging suite (Compumedics Neuroscan).

Recording electrodes (Ambu Neuroline disposable Ag/AgCl cup electrodes) were positioned at Cz, CPz, C3, C4, FCz, Oz, T3 and T4, according to the modified international 10/20 electrode placement system. Reference and ground electrodes were placed at Fz and on the forehead respectively. A reduced electrode montage was applied in 8 infants but activity was always recorded at the Cz electrode. In 10 infants the recordings were acquired with reference to Fpz and re-referenced post-acquisition to Fz. Impedance was minimised by gentle rubbing of the skin with EEG preparation gel (NuPrep gel, D.O. Weaver and Co.) and conductance paste (Elefix EEG paste, Nihon Kohden) was used to optimise skin contact. Electrodes were held in place with an elasticated net cap.

Bipolar EMG electrodes (Ambu Neuroline 700 solid gel surface electrodes) were applied to the biceps femoris of the infant's leg ipsilateral to the site of stimulation.

The lance was time-locked to the EEG and EMG recordings using an event-detection interface and accelerometer [S2]. In 10 out of the 40 infants, the events were time-locked using a microphone secured to the lancet, with the audio recording directly linked to the electrophysiological recordings. There was no significant difference between the latencies to the evoked response recorded time-locked using the microphone and those of aged-matched infants time-locked using the accelerometer ($p = 0.95$, Wilcoxon signed rank test).

Threshold for Nociceptive-Specific Brain Activity

For each infant 10 background epochs were extracted. The EEG traces were Woody filtered, with a maximum jitter of ± 50 ms, in the region of 400 - 700 ms, by identifying the maximum correlation with the component of nociceptive-specific brain activity. The nociceptive-specific component was then projected onto the background data to identify a weight for each individual trace. From these values, the distribution of background weights was obtained, indicating the background noise levels within the data. The weights of each individual's response to heel lance were then compared to the distribution of background weights (formed from all the infant's data). A nociceptive-specific response was defined to have occurred if the weight of the evoked potential was greater than a threshold set at 90 % of the distribution of background weights (0.14) [S3].

Determination of EMG Characteristics

In order to accurately identify the start and end times of the reflex a novel algorithm was developed. This defines the start and end times of the reflex withdrawal by first low pass filtering the signal at 5 Hz, and then smoothing the filtered signal further by averaging the signal across moving windows of length 250 ms (see Figure S3). The start point of the reflex withdrawal was defined as the point at which the magnitude of the differential of the smoothed signal went above a threshold (θ_s – see below) and the end point of the reflex withdrawal was defined as the point at which the differential falls below another

threshold (θ_e). The differential was used as this allows for the infant's muscle activity to settle at a level above the baseline after the reflex withdrawal, reflecting a change in muscle tone.

Let $f(t)$ be the smoothed signal and the differential be given by $g(t) = \frac{df(t)}{dt}$.

Then the start time τ_s is defined as the first time point such that:

$$g(\tau_s + t) > \theta_s \ \forall\, t \in [0,100], \tau_s > 0, \tau_s < 3000$$

where

$$\theta_s = \max\{0.0045, 5\sigma(b)\}$$

(b is the baseline of the differentiated signal, i.e. $b = g(t), \forall\, t \in [-2250, -250]$, times given in ms, and σ denotes the standard deviation).

The end point τ_e is defined as the first time point such that:

$$|g(\tau_e + t)| < \theta_e \ \forall\, t \in [0,250], \tau_e > \tau_r, \tau_e < 14{,}000$$

where

$$\theta_e = \max\{0.0025, \sigma(b)\},$$
$$g(\tau_r + t) < \theta_r \ \forall\, t \in [0,100], \tau_r > \tau_s,$$
$$\theta_r = -\theta_e$$

The duration of the reflex withdrawal was defined as the difference between the end and start times. The magnitude of the reflex withdrawal was defined as the area under the smoothed curve between the start and end times. The peak latency was defined as the time from the heel lance to the maximum of the smoothed curve between the start and end times (see Figure S3).

Supplemental References

[S1] Papile, L.A., Burstein, J., Burstein, R., and Koffler, H. (1978). Incidence and evolution of subependymal and intraventricular hemorrhage: a study of infants with birth weights less than 1,500 gm. J Pediatr, *92*, 529-534.

[S2] Worley, A., Fabrizi, L., Boyd, S., and Slater, R. (2012). Multi-modal pain measurements in infants. J Neurosci Methods, *205*, 252-257.

[S3] Fabrizi, L., Slater, R., Worley, A., Meek, J., Boyd, S., Olhede, S., and Fitzgerald, M. (2011). A shift in sensory processing that enables the developing human brain to discriminate touch from pain. Curr Biol, *21*, 1552-1558.
