# Supplementary material for: Changing Balance of Spinal Cord Excitability and Nociceptive Brain Activity in Early Human Development
Source: Curr Biol. 2016 Aug 8;26(15):1998–2002. doi: 10.1016/j.cub.2016.05.054 (PMC4985558; doi:10.1016/j.cub.2016.05.054)
Supplement: Document S2. Article plus Supplemental Information [file mmc2.pdf]

Report

Current Biology

Changing Balance of Spinal Cord Excitability and Nociceptive Brain Activity in Early Human Development

Graphical Abstract

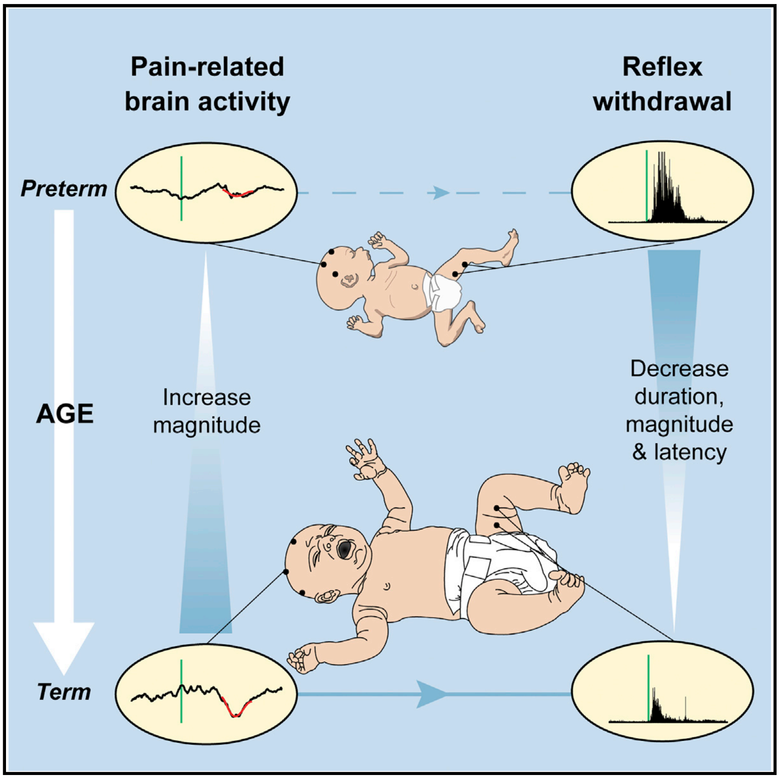

Highlights

- Noxious-evoked brain activity increases in magnitude across the preterm period
- Maturation of nociceptive brain activity coincides with reflex activity refinement
- This may relate to the emergence of top-down inhibition in human infants

Authors

Caroline Hartley, Fiona Moultrie, Deniz Gursul, Amy Hoskin, Eleri Adams, Richard Rogers, Rebeccah Slater

Correspondence

rebeccah.slater@paediatrics.ox.ac.uk

In Brief

Hartley et al. demonstrate that the maturation of nociceptive brain activity is concomitant with the refinement of reflex withdrawal in human infants. This change in balance of spinal cord excitability and nociceptive brain activity may arise due to the emergence of top-down inhibitory pathways during early human development.

CrossMark

Hartley et al., 2016, Current Biology *26*, 1998–2002
August 8, 2016 © 2016 The Author(s). Published by Elsevier Ltd.
http://dx.doi.org/10.1016/j.cub.2016.05.054

CellPress

Current Biology
Report

Changing Balance of Spinal Cord Excitability and Nociceptive Brain Activity in Early Human Development

Caroline Hartley,[1,3] Fiona Moultrie,[1,3] Deniz Gursul,[1] Amy Hoskin,[1] Eleri Adams,[1] Richard Rogers,[2] and Rebeccah Slater[1,*]
[1]Department of Paediatrics, University of Oxford, Oxford OX3 9DU, UK
[2]Nuffield Department of Anaesthetics, John Radcliffe Hospital, Oxford OX3 9DU, UK
[3]Co-first author
*Correspondence: rebeccah.slater@paediatrics.ox.ac.uk
http://dx.doi.org/10.1016/j.cub.2016.05.054

SUMMARY

In adults, nociceptive reflexes and behavioral responses are modulated by a network of brain regions via descending projections to the spinal dorsal horn [1]. Coordinated responses to noxious inputs manifest from a balance of descending facilitation and inhibition. In contrast, young infants display exaggerated and uncoordinated limb reflexes [2]. Our understanding of nociceptive processing in the infant brain has been advanced by the use of electrophysiological and hemodynamic imaging [3–6]. From approximately 35 weeks' gestation, nociceptive-specific patterns of brain activity emerge [7], whereas prior to this, non-specific bursts of activity occur in response to noxious, tactile, visual, and auditory stimulation [7–10]. During the preterm period, refinement of spinal cord excitability is also observed: reflex duration shortens, response threshold increases, and improved discrimination between tactile and noxious events occurs [2, 11, 12]. However, the development of descending modulation in human infants remains relatively unexplored. In 40 infants aged 28–42 weeks' gestation, we examined the relationship between nociceptive brain activity and spinal reflex withdrawal activity in response to a clinically essential noxious procedure. Nociceptive-specific brain activity increases in magnitude with gestational age, whereas reflex withdrawal activity decreases in magnitude, duration, and latency across the same developmental period. By recording brain and spinal cord activity in the same infants, we demonstrate that the maturation of nociceptive brain activity is concomitant with the refinement of noxious-evoked limb reflexes. We postulate that, consistent with studies in animals, infant reflexes are influenced by the development of top-down inhibitory modulation from maturing subcortical and cortical brain networks.

RESULTS AND DISCUSSION

Magnitude of Nociceptive-Specific Brain Activity Increases with Gestational Age

Electroencephalogram (EEG) responses to a clinically required heel lance were recorded in infants aged between 28 and 42 weeks' gestation. Nociceptive-specific brain activity (defined as evoked activity distinct from that evoked by non-noxious tactile stimulation—see the Experimental Procedures) was identified at the Cz electrode in 19 out of 36 infants and was not present in any of the infants who were younger than 32 weeks' gestation (n = 7). In infants who were greater than 32 weeks' gestation, nociceptive-specific brain activity was identified in 66% of infants (19 out of 29), and the magnitude of this activity significantly increased with gestational age (Figure 1; $p = 0.031$, regression coefficient $\beta = 0.024$, $n = 19$). This could not be accounted for by postnatal age, estimated cumulative prior pain exposure, or previous diagnosis of postnatal infection, which were included within the statistical model (Table S1).

Figure 1 includes four example EEG responses across a range of gestational ages. In the youngest infants, nociceptive-specific brain activity did not occur and non-modality specific bursts of activity [7–10], known as delta brushes, could be identified. This supports previous observations that the likelihood of evoking nociceptive-specific patterns of brain activity increases with gestational age and that the brain's ability to discriminate between tactile and noxious inputs emerges at approximately 35 weeks' gestation [7].

The emergence of nociceptive-specific brain activity during the preterm period coincides with the disappearance of the subplate and the formation of direct thalamocortical connections [7, 13]. Given that key clinical factors and demographic characteristics have been controlled for, the increase in magnitude of the evoked activity is likely to represent a normal maturational process. It is plausible, for example, that it may reflect the strengthening and elaboration of thalamocortical connectivity that occurs during this developmental period [14].

Here we focus on activity at the Cz electrode as nociceptive-specific brain activity has previously been identified and characterized at this electrode site [6, 7]. In the absence of this activity, responses with similar latency and morphology did not occur at other electrode sites (Figure S1). This does not rule out the likelihood that nociceptive activity could be recorded at other

 © 2016 The Author(s). Published by Elsevier Ltd.
This is an open access article under the CC BY license (http://creativecommons.org/licenses/by/4.0/).

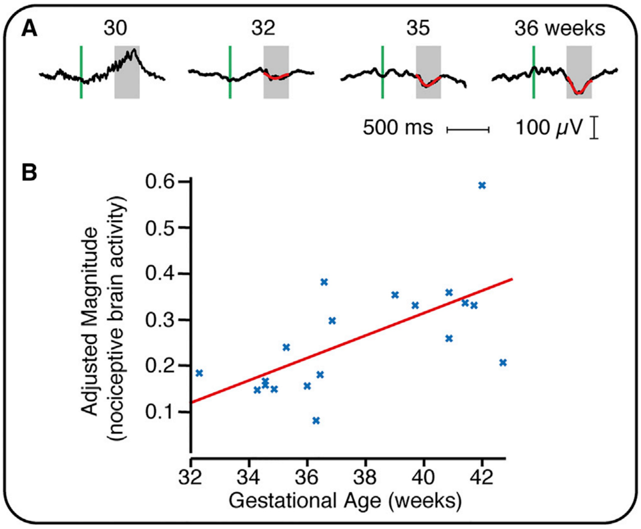

Figure 1. Relationship between Nociceptive-Specific Brain Activity and Gestational Age

(A) Four example EEG traces (black lines) from infants at different gestational ages. The green vertical line indicates the point of stimulation, and nociceptive-specific activity is overlaid in red.

(B) The magnitude of the nociceptive-specific brain activity significantly increased with gestational age (n = 19). The red line indicates the regression. No significant effect was observed with postnatal age, estimated cumulative prior pain exposure, and previous diagnosis of postnatal infection (Table S1). The magnitude is shown adjusted for these variables and only includes data where nociceptive-specific brain activity has been identified.

See also Figure S1.

electrode sites, but it provides a useful way of quantifying the maturation of nociceptive processing in the developing infant brain. Furthermore, activity recorded at the Cz site is likely to include contributions from numerous brain regions, including the primary and secondary somatosensory cortices, the anterior cingulate cortex, and the insular cortices [15].

Nociceptive Reflex Withdrawal Activity Becomes More Refined with Gestational Age

Reflex withdrawal activity was recorded in the limb ipsilateral to the site of stimulation by placement of electromyography (EMG) electrodes over the biceps femoris. A novel algorithm that defines the start and end points of infant reflex withdrawal was used to identify the changing characteristics of reflex activity across development (see the Experimental Procedures). Figure 2A includes four example EMG traces across a range of gestational ages. We observed that increasing gestational age was associated with a significant decrease in the duration (Figure 2B; $p = 0.039$, $\beta = -0.29$, n = 32), magnitude (Figure 2C; $p = 0.001$, $\beta = -0.02$), and latency to the peak reflex response (Figure 2D; $p = 0.002$, $\beta = -0.15$; see also Table S1), representing a decrease in magnitude of 20 mV per week and a decrease in duration of 0.3 s per week. This refinement in reflex withdrawal activity is consistent with previous observations in both animals and human infants [2, 16].

Infant limb reflexes are exaggerated and disorganized. In young rat pups, reflexes have longer duration, higher magnitude, and lower response thresholds compared with adult animals [12, 16]. Moreover, rat pups are more likely to inappropriately flick their tail toward a noxious stimulus than away from it [17]. In preterm infants, reflexes are prolonged, thresholds lie within the tactile range, and bilateral responses occur after unilateral stimulation [2, 11, 18]. The lower reflex withdrawal threshold likely allows for activity-dependent development of nociceptive processing [2, 17, 19]. In addition, reflex maturation is dependent on supraspinal activity, with neonatal spinal cord transection resulting in disorganized and exaggerated reflexes in the adult animal [20]. In young rat pups, the cutaneous receptive fields of dorsal horn cells are larger, mature over the first few weeks of postnatal life [21], and contribute to the exaggerated reflex activity observed in young animals [22–24].

Refinement of Reflex Withdrawal Activity Is Related to Maturation of the Nociceptive-Specific Brain Activity

Finally, we investigated the development of the relationship between nociceptive-specific brain activity and reflex withdrawal activity. When considering the emergence of the nociceptive-specific brain activity and the diminution of the reflex activity, the relative proportion of nociceptive-specific brain activity to reflex withdrawal activity within individual infants significantly increases with gestational age (Figure 3; $p = 0.024$, $\beta = 0.054$, n = 29; see also Table S1). There was no significant effect of postnatal age, estimated cumulative prior pain exposure, or previous diagnosis of postnatal infection (see Table S1). Noxious input in younger infants elicits more prolonged reflex withdrawal activity but comparatively less nociceptive-specific brain activity compared with older infants. The presence of more mature nociceptive-specific brain activity in older infants is concomitant with a more acute reflexive response (Figure 3). All electrophysiological recordings in which both EMG and EEG activity were artifact-free are included in Figure S2.

Independent observations describing the reduction in reflex withdrawal activity [2] and the emergence of nociceptive-specific brain activity [7] across the preterm period have been reported. In term infants, the cortical and subcortical brain regions activated by noxious stimulation are similar to those seen in adults [4], and there is a clear correlation between the magnitude of noxious-evoked brain activity and spinally mediated reflex withdrawal activity [25]. However, the emerging relationship between spinally mediated reflexes and nociceptive brain activity that underpins the perception and expression of pain has not previously been investigated in premature infants. The change in balance of nociceptive-specific brain activity and spinally mediated reflex activity suggests that the maturation of nociceptive brain activity may facilitate the inhibitory modulation of spinal nociceptive circuitry. This is consistent with animal studies; in rat pups, the descending control system is immature and the drive from the rostral ventral medulla is predominantly excitatory [26]. This contributes toward the exaggerated and uncoordinated reflex activity observed in young animals [12, 17, 22, 26, 27]. Descending inhibitory influences develop over the first few postnatal weeks, and from the fourth postnatal week more mature cerebral processing of nociceptive input can dampen spinally mediated reflex withdrawal activity [26]. Additionally, it has recently been proposed that the descending facilitation of spinal nociception that is observed in the first few postnatal

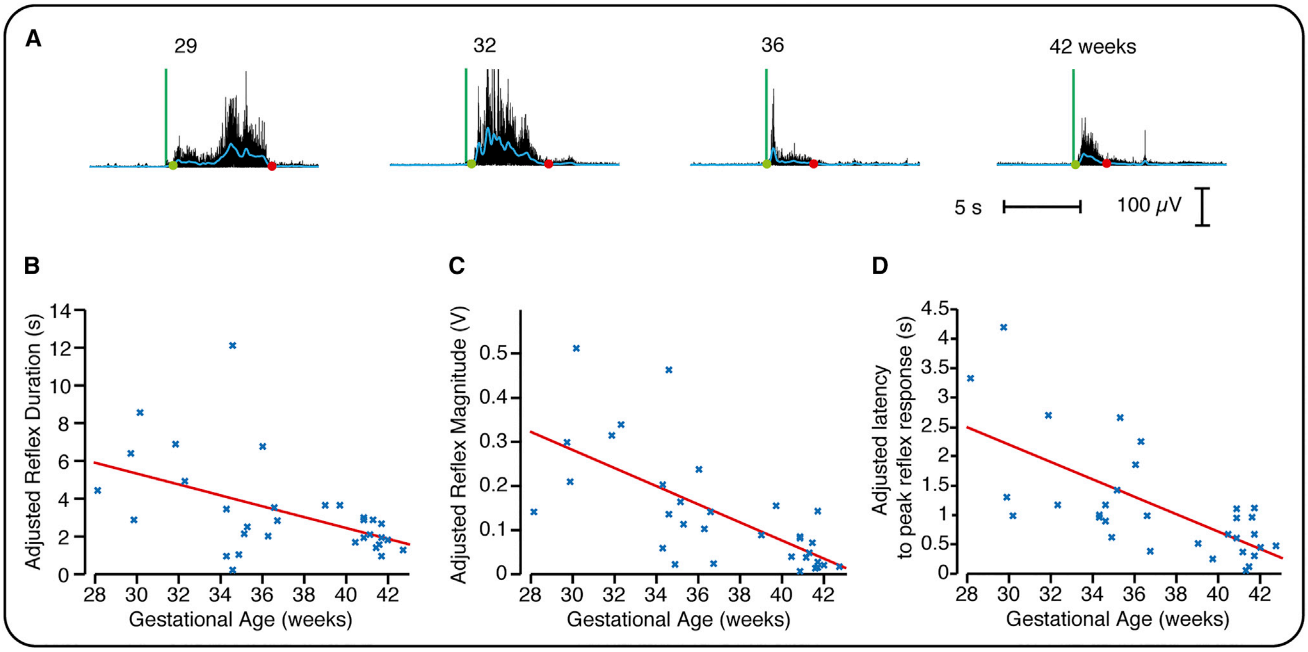

Figure 2. Refinement of Reflex Withdrawal Activity

(A) Four example EMG traces (black lines) from infants at different gestational ages. The green vertical line indicates the point of stimulation, and the blue line represents the smoothed reflex withdrawal activity. The start and end points are identified by the green and red dots, respectively (see also Figure S3).

(B–D) The duration (B), magnitude (C), and latency to the peak of the reflex withdrawal activity (D) significantly decreased with gestational age (n = 32). No significant effect was observed with postnatal age, estimated cumulative prior pain exposure, and previous diagnosis of postnatal infection (Table S1). The data are shown adjusted for these variables.

weeks is likely to be generated by spontaneous brainstem activity that is independent of sensory input [28].

In adults, top-down connections play a key role in modulating pain perception [1]. This involves an extensive network of brain regions that include the anterior cingulate cortex, insular cortices, and brainstem [1]. In full-term infants, these brain regions are actively involved in processing nociceptive input [4] and may contribute toward the generation of the electrophysiological nociceptive activity characterized here [15]. We postulate that the maturation of cortical networks, which is reflected here as an increase in the magnitude of nociceptive-specific brain activity, contributes to the emergence of top-down inhibitory modulation of spinal nociceptive circuitry. Analysis of fMRI data should be used to explore how regional brain development modulates pain-related behavior in human infants.

Conclusions

In conclusion, we demonstrate that across the early developmental period from 28–42 weeks' gestation, the maturation of noxious-evoked brain activity coincides with the refinement of reflex withdrawal activity. We postulate that the change in balance of nociceptive-specific brain activity and reflex withdrawal with gestational age is driven by the development of supraspinal processing, which results in the modulation of spinally mediated reflex withdrawal. This study provides new insights into the functional development of neural pathways that underlie human pain behavior and represents an important step toward translation of laboratory animal data.

EXPERIMENTAL PROCEDURES

Subjects

40 infants were recruited between May 2012 and June 2015 from the Neonatal and Maternity Units of the John Radcliffe Hospital, Oxford. Infants were aged between 28 and 42 weeks' gestation at time of study and aged 33 days or less. Further infant demographics are listed in Tables 1 and S2. (See "Infant Eligibility" in the Supplemental Experimental Procedures.) Ethical approval (National Research Ethics Service) was obtained, and informed written parental consent was gained prior to each study. The study was carried out in accordance with the standards set by the Declaration of Helsinki and Good Clinical Practice guidelines.

Experimental Protocol

All heel lances performed in the study were clinically required as part of the infant's medical care. EMG and EEG activity was recorded during a background rest period and during a clinically required heel lance (see "EEG and EMG Acquisition" in the Supplemental Experimental Procedures).

Analysis

EEG

EEG was filtered 0.5–70 Hz, with a notch filter at 50 Hz. 1500 ms epochs were extracted with 500 ms before the stimulus and traces were baseline corrected to the pre-stimulus mean. A total of four infants were rejected from EEG analysis because epochs contained gross movement or signal artifacts (for example, one epoch was rejected due to repetitive artifacts caused by the infant's respiratory support).

Nociceptive-specific brain activity is known to be evoked at the Cz electrode [6]. For calculation of the magnitude of the activity, the component of nociceptive-specific brain activity (defined in an independent sample of term infants [25]) was projected onto the data using singular value decomposition [25]. This enabled the weights (magnitude) of the evoked nociceptive-specific brain activity to be determined in this study. The EEG traces were Woody filtered,

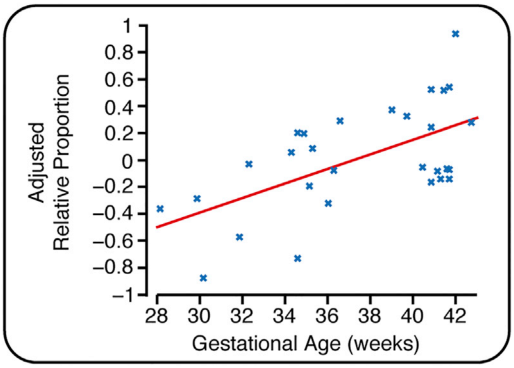

Figure 3. The Relationship between Nociceptive-Specific Brain Activity and Reflex Withdrawal Activity with Gestational Age

The relative proportion of brain and spinal cord activity for each infant plotted against gestational age (n = 29; where brain activity and reflex activity were both recorded without artifact). The values of the relative proportion are limited between −1 and 1, where −1 indicates maximal reflex withdrawal (within the population) with no concomitant nociceptive-specific brain activity and 1 indicates maximal nociceptive-specific brain activity with no concomitant reflex withdrawal (see the Experimental Procedures). See also Figure S2 and Table S1.

with a maximum jitter of ±50 ms, in the region of 400–700 ms after the stimulus, by identifying the maximum correlation with the component of nociceptive-specific brain activity. The weights of the nociceptive-specific component (which are a reflection of the magnitude of the nociceptive-specific brain activity within an individual response) were then calculated in the region 400–700 ms after the stimulus at the Cz electrode. A threshold to define whether nociceptive-specific brain activity was present was set by comparison of the weights of the evoked activity to the weights generated from background brain activity (see "Threshold for Nociceptive-Specific Brain Activity" in the Supplemental Experimental Procedures).

EMG

Epochs were extracted from 15 s before to 15 s after the stimulus, and the signals were filtered between 10 and 500 Hz with a notch filter at 50 Hz (and harmonics) and rectified. Epochs were rejected due to high signal levels from high impedances and electrocardiogram (ECG) artifacts. For accurate identification of the start and end times of the reflex, a novel algorithm was developed (see "Determination of EMG Characteristics" in the Supplemental Experimental Procedures and Figure S3). Five infants were excluded from EMG analysis due to artifact. A further three infants were excluded as in two cases no end point was identified by the algorithm and in one case no reflex withdrawal could be identified.

Comparison of Brain Activity and Reflex Withdrawal

The maturation of brain activity and reflex withdrawal was compared by examining the relative proportion of the two signals across the gestational age range. This was determined by first calculating the proportion of each signal for each infant. This was defined as the signal normalized by the maximal signal (across all infants). So for each infant, the duration of the reflex withdrawal was divided by the maximum duration across the population, and the weight of the nociceptive-specific brain activity was divided by the maximum weight across the population. Below-threshold brain activity responses were set to 0 as the evoked activity was not greater than the levels of spontaneous activity observed in the background data. The relative proportion was then calculated as the difference in the proportion of nociceptive-specific brain activity and the proportion of reflex withdrawal. This gives a value between −1 and 1, where −1 indicates a maximal reflex withdrawal coupled with no nociceptive-specific brain activity and 1 indicates maximal nociceptive-specific brain activity with no reflex withdrawal.

Cumulative Prior Pain Exposure and Previous Diagnosis of Postnatal Infection

To provide an estimate of cumulative prior pain exposure, we retrospectively reviewed each infant's electronic and paper clinical records. The total number of aspirations (oropharyngeal or endotracheal) and tissue-damaging procedures performed for blood taking (including heel lances, venepuncture, and intravenous cannulations) from time of birth to time of study were documented. These procedures were chosen as they feature among the top six most common painful procedures performed in the first 2 weeks of life in infants receiving intensive care, as estimated by a large multicenter prospective study [29], and are well documented by the clinical team, facilitating retrospective review.

Infants with a previous diagnosis of postnatal infection were established by review of clinical records to identify infants who had received treatment for suspected culture-negative sepsis.

Statistical Analysis

Statistical analysis was performed in MATLAB R2014b (MathWorks). Linear regression analysis was conducted with gestational age at study, postnatal age, estimated cumulative prior pain exposure, and previous diagnosis of postnatal infection included as independent variables. Normality of the residuals was confirmed using Q-Q plots. Regression figures are presented with the dependent variable adjusted for postnatal age, estimated cumulative prior pain exposure, and previous diagnosis of postnatal infection.

Table 1. Description of Infant Demographics

Demographic Details	Values
Gestational age at birth (weeks)—median (IQR)	34.4 (29.6–40.6)
Gestational age at time of study (weeks)—median (IQR)	36.4 (33.3–40.9)
Postnatal age at time of study (days)—mean (SD)	12.1 (11.1)
Birth weight (g)—median (IQR)	2,194 (1,538–3,627)
Weight at study (g)—median (IQR)	2,325 (1,620–3,627)
Male infants (%)	20 (50)
Multiple gestation infants (%)	7 (18)
Spontaneous vaginal deliveries (%)	18 (45)
Assisted/caesarian deliveries (%)	22 (55)
Apgar score at 1 min—mean (SD)	7.2 (2.6)
Apgar score at 5 min—mean (SD)	9.1 (1.4)
Infants admitted to NICU (%)	23 (58)
Infants ventilated during admission (%)	9 (23)
Days of ventilation—mean (SD)	4.8 (6.4)
Estimated cumulative prior pain exposure—median (IQR)	8 (3.8–28)
Infants with grade I IVH (%)	3 (7)
Infants with history of previous surgery (%)	2 (5)
Infants with a previous diagnosis of postnatal infection (%)	30 (75)
Infants with history of necrotizing enterocolitis (%)	2 (5)

IQR, interquartile range; NICU, neonatal intensive care unit; IVH, intraventricular hemorrhage. See also Table S2.

SUPPLEMENTAL INFORMATION

Supplemental Information includes Supplemental Experimental Procedures, three figures, and two tables and can be found with this article online at http://dx.doi.org/10.1016/j.cub.2016.05.054.

AUTHOR CONTRIBUTIONS

R.S., F.M., and E.A. designed the study; F.M., C.H., and A.H. performed the experiments; C.H., F.M., R.S., and D.G. analyzed the data; C.H., F.M., and

R.S. wrote the paper; and R.S., C.H., F.M., D.G., R.R., A.H., and E.A. made a significant contribution to data interpretation. All authors critically revised the manuscript.

ACKNOWLEDGMENTS

This work was funded by the Wellcome Trust. Fiona Moultrie is an NIHR Academic Clinical Fellow. The authors would like to thank Ravi Poorun, Gabriela Schmidt Mellado, Sezgi Goksan, Faith Emery, and Gabrielle Green for help with data collection and Charlotte Moultrie for the Graphical Abstract design. We would also like to thank the children who took part in this study.

Received: February 24, 2016
Revised: April 25, 2016
Accepted: May 24, 2016
Published: June 30, 2016

REFERENCES

1. Bingel, U., and Tracey, I. (2008). Imaging CNS modulation of pain in humans. Physiology (Bethesda) *23*, 371–380.
2. Cornelissen, L., Fabrizi, L., Patten, D., Worley, A., Meek, J., Boyd, S., Slater, R., and Fitzgerald, M. (2013). Postnatal temporal, spatial and modality tuning of nociceptive cutaneous flexion reflexes in human infants. PLoS ONE *8*, e76470.
3. Bartocci, M., Bergqvist, L.L., Lagercrantz, H., and Anand, K.J. (2006). Pain activates cortical areas in the preterm newborn brain. Pain *122*, 109–117.
4. Goksan, S., Hartley, C., Emery, F., Cockrill, N., Poorun, R., Moultrie, F., Rogers, R., Campbell, J., Sanders, M., Adams, E., et al. (2015). fMRI reveals neural activity overlap between adult and infant pain. eLife *4*, e06356.
5. Slater, R., Cantarella, A., Gallella, S., Worley, A., Boyd, S., Meek, J., and Fitzgerald, M. (2006). Cortical pain responses in human infants. J. Neurosci. *26*, 3662–3666.
6. Slater, R., Worley, A., Fabrizi, L., Roberts, S., Meek, J., Boyd, S., and Fitzgerald, M. (2010). Evoked potentials generated by noxious stimulation in the human infant brain. Eur. J. Pain *14*, 321–326.
7. Fabrizi, L., Slater, R., Worley, A., Meek, J., Boyd, S., Olhede, S., and Fitzgerald, M. (2011). A shift in sensory processing that enables the developing human brain to discriminate touch from pain. Curr. Biol. *21*, 1552–1558.
8. Chipaux, M., Colonnese, M.T., Mauguen, A., Fellous, L., Mokhtari, M., Lezcano, O., Milh, M., Dulac, O., Chiron, C., Khazipov, R., and Kaminska, A. (2013). Auditory stimuli mimicking ambient sounds drive temporal “delta-brushes” in premature infants. PLoS ONE *8*, e79028.
9. Colonnese, M.T., Kaminska, A., Minlebaev, M., Milh, M., Bloem, B., Lescure, S., Moriette, G., Chiron, C., Ben-Ari, Y., and Khazipov, R. (2010). A conserved switch in sensory processing prepares developing neocortex for vision. Neuron *67*, 480–498.
10. Milh, M., Kaminska, A., Huon, C., Lapillonne, A., Ben-Ari, Y., and Khazipov, R. (2007). Rapid cortical oscillations and early motor activity in premature human neonate. Cereb. Cortex *17*, 1582–1594.
11. Andrews, K., and Fitzgerald, M. (1999). Cutaneous flexion reflex in human neonates: a quantitative study of threshold and stimulus-response characteristics after single and repeated stimuli. Dev. Med. Child Neurol. *41*, 696–703.
12. Fitzgerald, M., Shaw, A., and MacIntosh, N. (1988). Postnatal development of the cutaneous flexor reflex: comparative study of preterm infants and newborn rat pups. Dev. Med. Child Neurol. *30*, 520–526.
13. Kostović, I., and Judas, M. (2010). The development of the subplate and thalamocortical connections in the human foetal brain. Acta Paediatr. *99*, 1119–1127.
14. Kostović, I., and Jovanov-Milosević, N. (2006). The development of cerebral connections during the first 20-45 weeks’ gestation. Semin. Fetal Neonatal Med. *11*, 415–422.
15. Garcia-Larrea, L., Frot, M., and Valeriani, M. (2003). Brain generators of laser-evoked potentials: from dipoles to functional significance. Neurophysiol. Clin. *33*, 279–292.
16. Fitzgerald, M., and Gibson, S. (1984). The postnatal physiological and neurochemical development of peripheral sensory C fibres. Neuroscience *13*, 933–944.
17. Waldenström, A., Thelin, J., Thimansson, E., Levinsson, A., and Schouenborg, J. (2003). Developmental learning in a pain-related system: evidence for a cross-modality mechanism. J. Neurosci. *23*, 7719–7725.
18. Andrews, K.A., Desai, D., Dhillon, H.K., Wilcox, D.T., and Fitzgerald, M. (2002). Abdominal sensitivity in the first year of life: comparison of infants with and without prenatally diagnosed unilateral hydronephrosis. Pain *100*, 35–46.
19. Koch, S.C., and Fitzgerald, M. (2013). Activity-dependent development of tactile and nociceptive spinal cord circuits. Ann. N Y Acad. Sci. *1279*, 97–102.
20. Levinsson, A., Luo, X.L., Holmberg, H., and Schouenborg, J. (1999). Developmental tuning in a spinal nociceptive system: effects of neonatal spinalization. J. Neurosci. *19*, 10397–10403.
21. Fitzgerald, M. (1985). The post-natal development of cutaneous afferent fibre input and receptive field organization in the rat dorsal horn. J. Physiol. *364*, 1–18.
22. Holmberg, H., and Schouenborg, J. (1996). Postnatal development of the nociceptive withdrawal reflexes in the rat: a behavioural and electromyographic study. J. Physiol. *493*, 239–252.
23. Fitzgerald, M. (2005). The development of nociceptive circuits. Nat. Rev. Neurosci. *6*, 507–520.
24. Fitzgerald, M., and Jennings, E. (1999). The postnatal development of spinal sensory processing. Proc. Natl. Acad. Sci. USA *96*, 7719–7722.
25. Hartley, C., Goksan, S., Poorun, R., Brotherhood, K., Mellado, G.S., Moultrie, F., Rogers, R., Adams, E., and Slater, R. (2015). The relationship between nociceptive brain activity, spinal reflex withdrawal and behaviour in newborn infants. Sci. Rep. *5*, 12519.
26. Hathway, G.J., Koch, S., Low, L., and Fitzgerald, M. (2009). The changing balance of brainstem-spinal cord modulation of pain processing over the first weeks of rat postnatal life. J. Physiol. *587*, 2927–2935.
27. Boucher, T., Jennings, E., and Fitzgerald, M. (1998). The onset of diffuse noxious inhibitory controls in postnatal rat pups: a C-Fos study. Neurosci. Lett. *257*, 9–12.
28. Schwaller, F., Kwok, C., and Fitzgerald, M. (2016). Postnatal maturation of the spinal-bulbo-spinal loop: brainstem control of spinal nociception is independent of sensory input in neonatal rats. Pain *157*, 677–686.
29. Carbajal, R., Rousset, A., Danan, C., Coquery, S., Nolent, P., Ducrocq, S., Saizou, C., Lapillonne, A., Granier, M., Durand, P., et al. (2008). Epidemiology and treatment of painful procedures in neonates in intensive care units. JAMA *300*, 60–70.

Current Biology, Volume *26*

Supplemental Information

Changing Balance of Spinal Cord Excitability and Nociceptive Brain Activity in Early Human Development

Caroline Hartley, Fiona Moultrie, Deniz Gursul, Amy Hoskin, Eleri Adams, Richard Rogers, and Rebeccah Slater

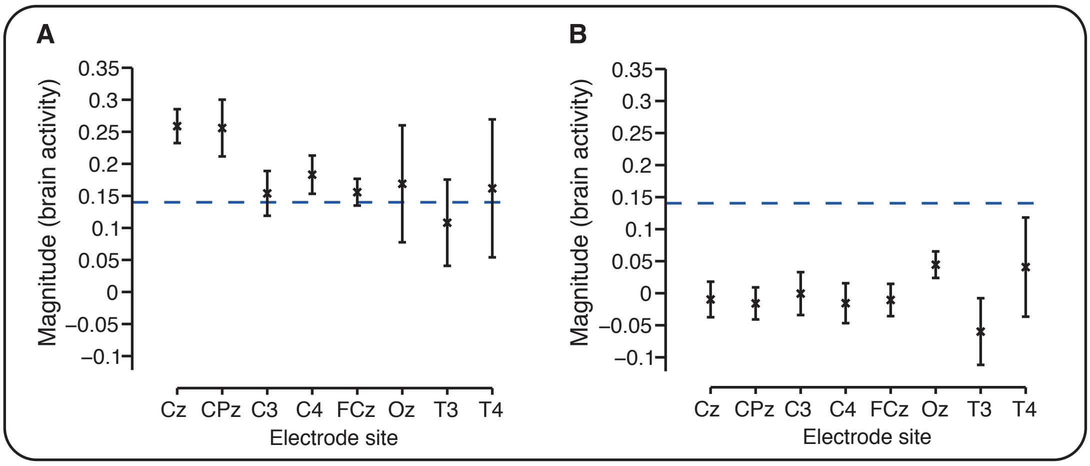

Figure S1: Magnitude of the response at other electrode sites, related to Figure 1. Magnitude of the nociceptive brain activity at each electrode site grouped according to whether nociceptive-specific activity was identified at Cz. (A) shows the average activity when nociceptive-specific activity was identified at Cz and (B) shows the average activity when nociceptive-specific activity was not identified at Cz. Error bars indicate the standard error of the mean. Blue dashed lines indicate the threshold for a nociceptive-specific response.

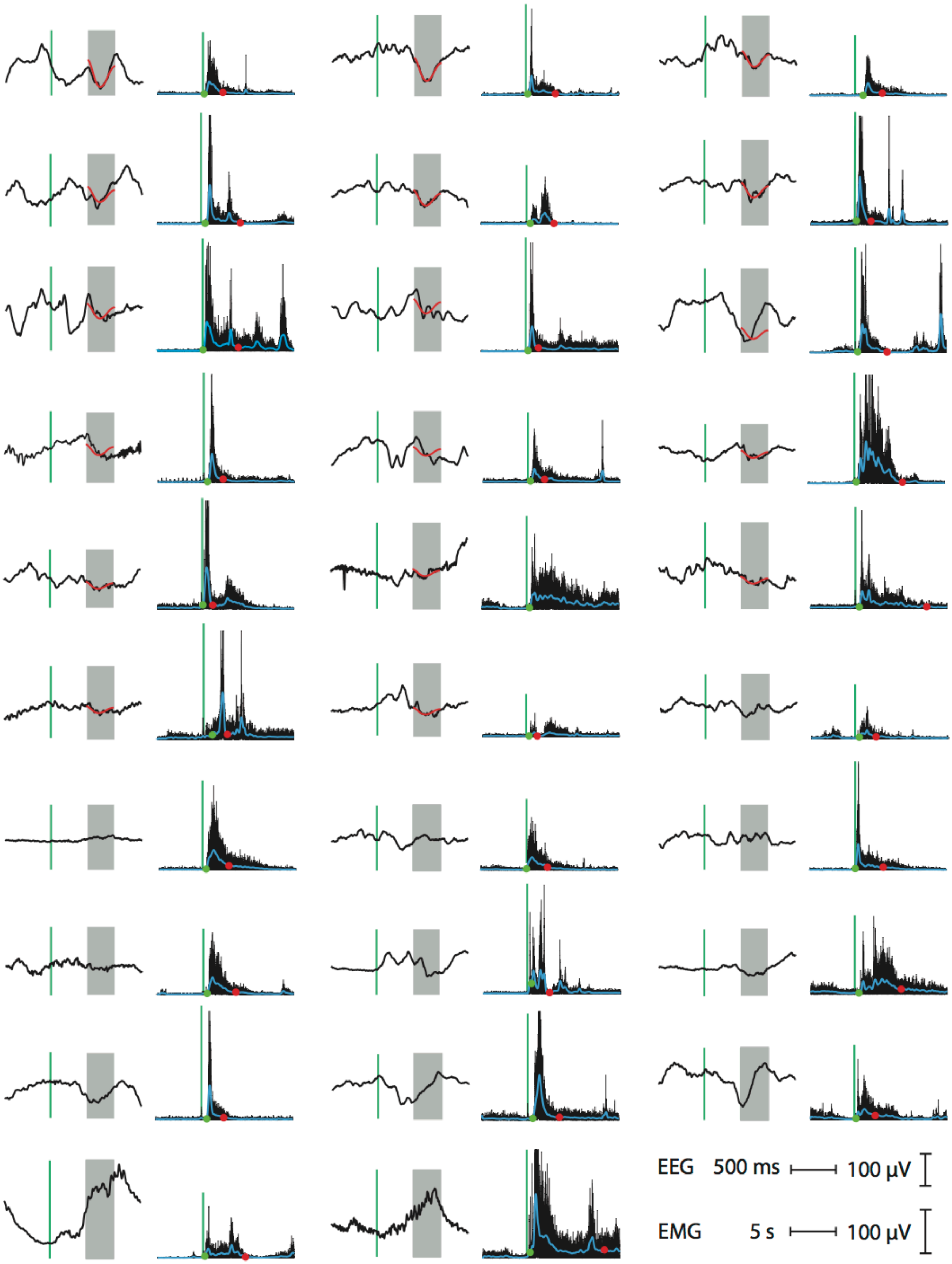

Figure S2: Brain activity and reflex withdrawal activity, related to Figure 3.
Individual infant EEG traces and EMG traces for all infants where artifact-free recordings were simultaneously recorded (3 infants are shown per row; EEG activity at the Cz electrode site is displayed on the left and EMG activity is displayed on the right; n=29). The point of stimulation is indicated by the

green vertical line. The grey box indicates the time region from 400 - 700 ms after stimulation. When nociceptive-specific responses were identified, the component of nociceptive-specific brain activity is overlaid in red. The raw EMG activity is overlaid with the smoothed EMG activity (blue line, see Supplemental Experimental Procedures). The green dot represents the start of the reflex and the red dot represents the end.

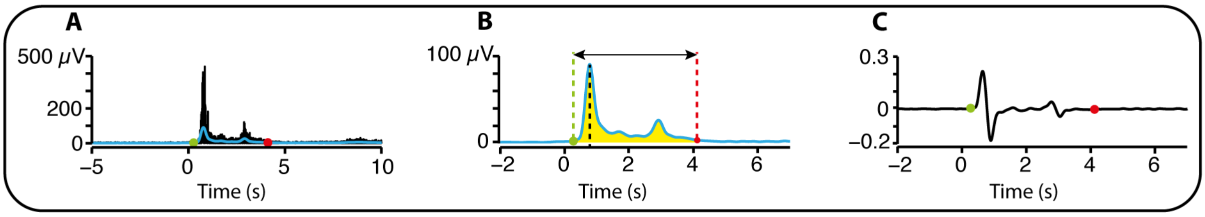

Figure S3: Novel algorithm used to characterise reflex withdrawal, related to Figure 2.
(A) Example reflex withdrawal recorded using EMG (activity shown in black). This activity is smoothed (blue line), and the start (green dot) and end times (red dot) are calculated using a novel algorithm.
(B) The smoothed EMG signal (blue, from A) is used to find the magnitude of the reflex withdrawal, given by the area under the curve between the start and end times (shaded yellow), the duration of the reflex withdrawal, defined as the time between the start and end points (indicated by the arrow), and the peak latency (time to the black dashed line). (C) The differential (black) of the smoothed signal (shown in blue in A, B), which is used to calculate the start (green dot) and end (red dot) times of the reflex withdrawal.

Dependent variables	Gestational Age (at time of study)			Postnatal Age			Previous diagnosis of postnatal infection			Estimated cumulative prior pain exposure		
	**β (CI)**	**β* (CI)**	**P**	**β (CI)**	**β* (CI)**	**P**	**β (CI)**	**β* (CI)**	**P**	**β (CI)**	**β* (CI)**	**P**
Magnitude of nociceptive-specific brain activity (n = 19)	0.024 (0.003 to 0.046)	0.67 (0.07 to 1.27)	**0.031***	0.0004 (-0.007 to 0.008)	0.041 (-0.66 to 0.74)	0.90	-0.092 (-0.23 to 0.041)	-0.80 (-1.95 to 0.35)	0.16	0.0008 (-0.002 to 0.004)	0.18 (-0.44 to 0.80)	0.54
Duration of reflex withdrawal (n = 32)	-0.29 (-0.56 to -0.016)	-0.50 (-0.98 to -0.027)	**0.039***	0.035 (-0.098 to 0.17)	0.15 (-0.43 to 0.73)	0.59	0.55 (-1.86 to 2.96)	0.22 (-0.75 to 1.20)	0.64	-0.028 (-0.071 to 0.015)	-0.33 (-0.84 to 0.18)	0.20
Magnitude of reflex withdrawal (n = 32)	-0.020 (-0.032 to -0.009)	-0.77 (-1.21 to -0.32)	**0.001***	-0.003 (-0.009 to 0.003)	-0.29 (-0.84 to 0.26)	0.28	0.019 (-0.087 to 0.12)	0.16 (-0.75 to 1.07)	0.72	-0.0006 (-0.003 to 0.001)	-0.15 (-0.63 to 0.33)	0.52
Peak latency of reflex withdrawal (n = 32)	-0.15 (-0.24 to -0.060)	-0.72 (-1.16 to -0.30)	**0.002***	-0.031 (-0.075 to 0.011)	-0.40 (-0.93 to 0.13)	0.14	0.27 (-0.51 to 1.05)	0.31 (-0.57 to 1.19)	0.48	0.006 (-0.008 to 0.020)	0.21 (-0.25 to 0.68)	0.35
Relative proportion of EEG to EMG activity (n = 29)	0.054 (0.008 to 0.10)	0.64 (0.09 to 1.18)	**0.024***	0.003 (-0.018 to 0.024)	0.088 (-0.56 to 0.73)	0.78	-0.29 (-0.70 to 0.12)	-0.79 (-1.91 to 0.32)	0.15	0.002 (-0.005 to 0.009)	0.16 (-0.40 to 0.72)	0.56

Table S1: Regression models, related to Figures 1, 2 and 3.
For each of the EEG and EMG characteristics (dependent variables) the regression coefficients (β); standardised regression coefficients (β*); confidence interval limits (CI) and p-values that are associated with the infant characteristics (gestational age, postnatal age, postnatal infection and estimated cumulative prior pain exposure) are given.

Subject	GA at study (weeks)	GA at birth (weeks)	Weight at birth (g)	Apgar score at 5 minutes	NICU	Mechanical ventilation (days)	Time since morphine administered (days)	IVH	PNI	Prior pain	Surgery
**1**	28.1	25.1	920	10	✓	1			✓	32	
**2**	29.7	28.3	855	6	✓		8		✓	16	
**3**	29.9	25.6	805	8	✓	5	28		✓	58	
**4**	30.1	28.9	640	8	✓					13	
**5**	30.9	29.0	1580	8	✓		12	✓		28	
**6**	31.6	27.3	720	8	✓	21	7	✓	✓	299	
**7**	31.7	29.0	1415	9	✓					17	
**8**	31.9	27.7	1315	10	✓	2	19		✓	124	
**9**	32.3	31.9	1580	8	✓				✓	6	
**10**	32.9	28.7	765	5	✓	1				35	
**11**	33.4	29.7	636	9	✓					41	
**12**	34.3	29.4	1390	4	✓	7	18		✓	111	Bowel surgery
**13**	34.3	32.0	2340	9	✓				✓	5	
**14**	34.6	31.0	1800	8	✓			✓	✓	8	
**15**	34.6	31.1	1610	10	✓				✓	14	
**16**	34.9	33.0	1820	9	✓	2	12		✓	28	
**17**	35.1	31.9	2020	8	✓	2	13		✓	42	Bowel surgery
**18**	35.3	31.9	1982	10	✓					14	
**19**	36.0	32.3	1626	10	✓				✓	16	
**20**	36.3	35.9	2360	10						5	
**21**	36.4	36.1	1910	10	✓					8	
**22**	36.6	33.0	2048	9	✓	2	20			35	
**23**	36.7	36.0	2440	10	✓					14	
**24**	36.9	36.1	3330	10	✓				✓	23	
**25**	39.0	38.9	3215	10					✓	2	
**26**	39.7	39.3	3595	10					✓	2	
**27**	40.4	40.1	5120	10					✓	4	
**28**	40.9	40.1	3400	10					✓	4	
**29**	40.9	40.3	3770	10					✓	5	
**30**	40.9	40.9	3500	10					✓	1	
**31**	41.0	40.6	3360	10					✓	3	
**32**	41.1	41.0	3925	10					✓	1	
**33**	41.3	41.1	3975	10					✓	1	
**34**	41.4	41.3	4645	9					✓	1	
**35**	41.6	40.7	3725	10					✓	4	
**36**	41.7	41.4	4220	10					✓	4	
**37**	41.7	41.6	3955	10					✓	1	
**38**	41.7	41.1	4140	10					✓	3	
**39**	42.0	41.7	4320	10					✓	1	
**40**	42.7	42.0	3464	8					✓	4	

Table S2: Individual infant demographics, related to Table 1.
GA – gestational age, NICU – indicates whether the infant was admitted to the neonatal intensive care unit, PNI –postnatal infection: indicates whether the infant had received treatment for suspected culture-negative sepsis prior to the study, prior pain – indicates the estimated cumulative prior pain exposure.

Supplemental Experimental Procedures

Infant Eligibility

Infants were not eligible for inclusion if they had documented neurological malformations or symptoms, genetic disorders, or a history of meningitis, culture-positive sepsis, or maternal substance abuse. Clinical cranial ultrasound reports were reviewed for all infants born at <32 weeks gestation or weighing <1500g at birth. Only infants with normal scans or a small, uncomplicated, unilateral, isolated subependymal haemorrhage (grade 1 IVH; [S1]) were included. At the time of study, all infants were haemodynamically stable, not requiring mechanical ventilation, and had not received analgesics or sedatives in the preceding seven days.

EEG and EMG Acquisition

Electrophysiological activity was acquired using the SynAmps RT 64-channel headbox and amplifiers (Compumedics Neuroscan), with a bandwidth from DC - 400 Hz and a sampling rate of 2 kHz, and recorded using CURRYscan7 neuroimaging suite (Compumedics Neuroscan).

Recording electrodes (Ambu Neuroline disposable Ag/AgCl cup electrodes) were positioned at Cz, CPz, C3, C4, FCz, Oz, T3 and T4, according to the modified international 10/20 electrode placement system. Reference and ground electrodes were placed at Fz and on the forehead respectively. A reduced electrode montage was applied in 8 infants but activity was always recorded at the Cz electrode. In 10 infants the recordings were acquired with reference to Fpz and re-referenced post-acquisition to Fz. Impedance was minimised by gentle rubbing of the skin with EEG preparation gel (NuPrep gel, D.O. Weaver and Co.) and conductance paste (Elefix EEG paste, Nihon Kohden) was used to optimise skin contact. Electrodes were held in place with an elasticated net cap.

Bipolar EMG electrodes (Ambu Neuroline 700 solid gel surface electrodes) were applied to the biceps femoris of the infant's leg ipsilateral to the site of stimulation.

The lance was time-locked to the EEG and EMG recordings using an event-detection interface and accelerometer [S2]. In 10 out of the 40 infants, the events were time-locked using a microphone secured to the lancet, with the audio recording directly linked to the electrophysiological recordings. There was no significant difference between the latencies to the evoked response recorded time-locked using the microphone and those of aged-matched infants time-locked using the accelerometer ($p = 0.95$, Wilcoxon signed rank test).

Threshold for Nociceptive-Specific Brain Activity

For each infant 10 background epochs were extracted. The EEG traces were Woody filtered, with a maximum jitter of ± 50 ms, in the region of 400 - 700 ms, by identifying the maximum correlation with the component of nociceptive-specific brain activity. The nociceptive-specific component was then projected onto the background data to identify a weight for each individual trace. From these values, the distribution of background weights was obtained, indicating the background noise levels within the data. The weights of each individual's response to heel lance were then compared to the distribution of background weights (formed from all the infant's data). A nociceptive-specific response was defined to have occurred if the weight of the evoked potential was greater than a threshold set at 90 % of the distribution of background weights (0.14) [S3].

Determination of EMG Characteristics

In order to accurately identify the start and end times of the reflex a novel algorithm was developed. This defines the start and end times of the reflex withdrawal by first low pass filtering the signal at 5 Hz, and then smoothing the filtered signal further by averaging the signal across moving windows of length 250 ms (see Figure S3). The start point of the reflex withdrawal was defined as the point at which the magnitude of the differential of the smoothed signal went above a threshold (θ_s – see below) and the end point of the reflex withdrawal was defined as the point at which the differential falls below another

threshold (θ_e). The differential was used as this allows for the infant's muscle activity to settle at a level above the baseline after the reflex withdrawal, reflecting a change in muscle tone.

Let $f(t)$ be the smoothed signal and the differential be given by $g(t) = \frac{df(t)}{dt}$.

Then the start time τ_s is defined as the first time point such that:

$$g(\tau_s + t) > \theta_s \; \forall \, t \in [0,100], \tau_s > 0, \tau_s < 3000$$

where

$$\theta_s = \max\{0.0045, 5\sigma(b)\}$$

(b is the baseline of the differentiated signal, i.e. $b = g(t), \forall \, t \in [-2250, -250]$, times given in ms, and σ denotes the standard deviation).

The end point τ_e is defined as the first time point such that:

$$|g(\tau_e + t)| < \theta_e \; \forall \, t \in [0,250], \tau_e > \tau_r, \tau_e < 14{,}000$$

where

$$\theta_e = \max\{0.0025, \sigma(b)\},$$
$$g(\tau_r + t) < \theta_r \; \forall \, t \in [0,100], \tau_r > \tau_s,$$
$$\theta_r = -\theta_e$$

The duration of the reflex withdrawal was defined as the difference between the end and start times. The magnitude of the reflex withdrawal was defined as the area under the smoothed curve between the start and end times. The peak latency was defined as the time from the heel lance to the maximum of the smoothed curve between the start and end times (see Figure S3).

Supplemental References

[S1] Papile, L.A., Burstein, J., Burstein, R., and Koffler, H. (1978). Incidence and evolution of subependymal and intraventricular hemorrhage: a study of infants with birth weights less than 1,500 gm. J Pediatr, *92*, 529-534.

[S2] Worley, A., Fabrizi, L., Boyd, S., and Slater, R. (2012). Multi-modal pain measurements in infants. J Neurosci Methods, *205*, 252-257.

[S3] Fabrizi, L., Slater, R., Worley, A., Meek, J., Boyd, S., Olhede, S., and Fitzgerald, M. (2011). A shift in sensory processing that enables the developing human brain to discriminate touch from pain. Curr Biol, *21*, 1552-1558.
